# Disease Model: a Simplified Approach for Analysis and Management of Human Error: A Quality Improvement Study

**DOI:** 10.1097/MD.0000000000000711

**Published:** 2015-04-17

**Authors:** Mohammad H. I. Ahmad-Sabry

**Affiliations:** From the Alexandria Faculty of Medicine, Alexandria University, Alexandria, Egypt.

## Abstract

During 6 weeks, we had 4 incidents of echocardiography machine malfunction. There were 3 in the operating room, which were damaged due to intravenous (IV) fluid spillage over the keyboard of the machine leading to burning of the keyboard electric connection, and 1 in the cardiology department, which was damagaed due to spillage of coffee on it.

The malfunction had an economic impact on the hospital (about $ 20,000) in addition to the nonavailability of the ultrasound (US) machine for the cardiac patient after the incident till the end of the case and for consequent cases till the fixation of the machine.

We undertook an analysis of the incidents using simplified approach. The first incident happened when changing an empty IV fluid bag for a full one led to spillage of some fluid onto the keyboard. The second incidence was due to the use of needle to depressurize a medication bottle for continuous IV drip, and the third event was due to disconnection of the IV set from the bottle during transfer of the patient from operation room to intensive care unit. The fundamental problem is of course that fluid is harmful to the US machine. In addition, the machines are in a position between the patient bed and anesthesia machine. This means that IV pulls are on each side of the patient bed, which makes the machine vulnerable to fluid spillage.

We considered a machine modification, to create a protective cover, but this was hindered by complexity of keyboard of the US machine, technical and financial challenges, and the time it would take to achieve. Second, we considered the creation of a protocol, with putting the machine in a position where no IV pulls are around and transferring the machine out of the room when transferring the patient will endanger the machine by the IV fluid. Third, changing of human behavior; to do this, we announced the protocol in our anesthesia conference to make it known to each and every one. We taught residents, fellows, and staff about the new protocol.

Our simplified approach was effective for the prevention of fluid spillage over the US machine.

## INTRODUCTION

Medical errors were first mentioned in Hammurabi's laws (1780 B.C.).^1^ Medical risks were evaluated as worse than many activities in both civilian and industrial activities with estimated 44,000 to 98,000 deaths annually.^2^ Economical impact was evaluated as up to $ 29 billion in the USA.^3^ Analgesics or pain medications are involved in up to 30% of drug-related adverse effects. The incidence of wrong drug administration appears to be similar comparing anesthesiologists in 3 subcontinents.^4^ Between 1996 and 2004, 27,971 claims were made by the Danish Patient Insurance Association covering all medical specialties, of which 1256 files (4.5%) were related to anesthesia. In 24 cases of deaths, the patient's death was considered to result from the anesthetic procedure: 4 deaths were related to airway management, 2 to ventilation management, 4 to central venous catheter placement, 4 as a result of medication errors, 4 from infusion pump problems, and 4 after complications from regional blockades.^5^

During 1 month, we had 4 incidents of echocardiography machine malfunction. There were 3 in the operating room, which were damaged due to intravenous (IV) fluid spillage over the keyboard of the machine leading to burning of the keyboard electric connection, and 1 in the cardiology department, which were damaged due to spillage of coffee on it. The question is the disease model as a way to simplify training of personnel to overcome the problem of human error that is encountered in our daily medical practice. Our hypothesis is that this simplified approach is effective in training health workers to understand, prevent, and manage human error problems.

The aim of this study is to evaluate our simplified approach to manage and prevent human error and consequently improve patient safety.

## METHODS

Approval of the ethical committee of the Alexandria Faculty of Medicine was obtained regarding our simplified approach. No name for the personnel involved in the 4 incidents was on records.

No funding agent was involved in our study.

To simplify the approach to human errors in a way close to the medical staff clinical thinking and practice, we approached the human error as a disease process that has predisposing factors and need both diagnosis and management for full understanding and prevention as well as follow-up.

The medical system is vulnerable to errors (predisposing factors) due to many reasons:

1. Complexity of the human body
2. Many and varied interactions with technology
3. Multiple caregivers and handoffs for care
4. Poor communications among caregivers
5. High acuity of illness or injury
6. Medical environment is prone to distraction
7. Need for rapid decision and time pressure
8. High volume and unpredictable patient flow
9. Exhaustion and short staffing

In spite of the use of simulators in anesthesia training, human errors still happen. Many researchers in human errors in medicine are comparing the medical error rates with the errors of aviation as a model to imitate. As there are many similarities between the aviation and anesthesia, there are more differences between the practice and subjects (patients vs airplanes). We need to put these differences in mind, while managing human errors.

### Environment of Safety (Prevention)

As there is predisposing factors for errors, there is a culture of human safety, which helps in the prevention and proper management for the condition:

1. Acknowledges high risk and error-prone nature of health care
2. Shared acceptance of responsibility for risk reduction
3. Open communication about safety concerns in nonpunitive environment and freedom of fear of reporting problems
4. Facilitates reporting of errors and safety concerns
5. Learns from errors and redesigns safer systems
6. Encourages accountability of patient safety
7. Ensures that organizational structures, processes, goals, and rewards are aligned with improving patient safety

### How to Act Toward Errors (Root Cause Analysis)

Drilling down to root cause may be difficult and uncomfortable; do not mistake the apparent cause for the root cause, resist the temptation to stop looking for the root cause, and resist premature actions. Treating only symptoms (the obvious or proximate cause) will only lead to short-term improvement but will not prevent recurrence.

### Diagnosis

Identify the factors of the case. Who? What? Where? When? Why?

Describe the process: as designed, as usually performed, and as performed in this case.

### Treatment

1. Machine/drug modification
2. Create protocols (standards and guidelines)
3. Change of human behavior

### Follow-Up (SMART)

1. Specific: specific goals must be put
2. Measurable: way for measurement of our goals must be recognized
3. Accountability: data collector and analysis must be accounted for
4. Report: a way for reporting the data must be implemented
5. Timeframes: timing for goal achievement needs to be planned

## RESULTS

We considered a machine modification, to create a protective cover, but this was hindered by the complexity of the keyboard of the ultrasound (US) machine, technical and financial challenges, and the time it would take to achieve. Second, we considered the creation of protocol, for putting the machine in a position where no IV pulls are around and transferring the machine out of the room in case of transferring the patient will endanger the machine by the IV fluid. Third, changing of human behavior; to do this, we announced the protocol in our anesthesia conference to make it known to each and every one. We provided teaching for residents, fellows, and other staff about this new protocol. No similar incident was reported in the next following 12 months.

## DISCUSSION

Reviewing the literature, we found a report of an incident of malfunction of the computer keyboard of the anesthesia machine due to humidity condensation.^6^ Malfunction of the machines has an economic impact on the hospital (about $ 20,000) in addition to the nonavailability of US machine for the cardiac patient after the incident till the end of the case and for consequent cases till the fixation of the machine. We undertook an analysis of the incidents. The first incident happened when changing an empty IV fluid bag for a full one led to spillage of some fluid on to the keyboard. The second incidence was due to the use of needle to depressurize a medication bottle for continuous IV drip, and the third event was due to disconnection of the IV set from the bottle during transfer of the patient from operation room to an intensive care unit. The fundamental problem is of course that fluid is harmful to the US machine. In addition, the machines are in a position between the patient bed and anesthesia machine. This means that IV pulls are on each side of the patient bed, which makes the machine vulnerable to fluid spillage. As complexity can be a challenge for dealing with human errors,^2^ we consider the disease model an ideal approach to simplify the management of human medical errors as it simplifies its understanding and management for medical personnel. In our knowledge, it is the first time to use this model to simplify training medical personnel to understand, manage, and prevent human errors in medicine. Limited to 1 error (fluid spillage over the US machine) is one of our limitations. The use of the approach in 1 institute and the 12 months period of observation may be another limitations. Our simplified approach was effective in the prevention of fluid spillage over the US machine. Other studies to evaluate this simplified model on other human medical errors can be done to validate its efficacy in other situations and different institutes.

## References

[1] Spiegel AD Hammurabi's managed health care—circa 1700 B.C. *Manag Care* 1997; 6:78–82. 10168637

[2] Richardson B Bromirski B Hayden A Implementing a safe and reliable process for medication administration. *Clin Nurse Spec* 2012; 26:169–176. 22504475 10.1097/NUR.0b013e3182503fbe

[3] Al-Assaf AF Bumpus LJ Carter D Preventing errors in healthcare: a call for action. *Hosp Top* 2003; 81:5–12. 15141846 10.1080/00185860309598022

[4] Kohn LT The Institute of Medicine report on medical error: overview and implications for pharmacy. *Am J Health Syst Pharm* 2001; 58:63–66. 11194137 10.1093/ajhp/58.1.63

[5] Hove LD Steinmetz J Christoffersen JK Analysis of deaths related to anesthesia in the period 1996–2004 from closed claims registered by the Danish Patient Insurance Association. *Anesthesiology* 2007; 106:675–680. 17413904 10.1097/01.anes.0000264749.86145.e5

[6] Ushijima K Yamada Y Ikuta Y An anesthesia machine malfunction due to dew condensation produced by abnormally high humidity in the electronically controlled panel [Article in Japanese]. *Masui* 1997; 46:406–408. 9095618

